# *Drosophila* Pheromone-Sensing Neurons Expressing the *ppk25* Ion Channel Subunit Stimulate Male Courtship and Female Receptivity

**DOI:** 10.1371/journal.pgen.1004238

**Published:** 2014-03-27

**Authors:** Vinoy Vijayan, Rob Thistle, Tong Liu, Elena Starostina, Claudio W. Pikielny

**Affiliations:** 1 Department of Genetics, Geisel School of Medicine at Dartmouth, Hanover, New Hampshire, United States of America; 2 Neuroscience Center, Geisel School of Medicine at Dartmouth, Hanover, New Hampshire, United States of America; 3 Department of Molecular and Cell Biology and Helen Wills Neuroscience Institute, University of California, Berkeley, Berkeley, California, United States of America; 4 Howard Hughes Medical Institute, University of California, Berkeley, Berkeley, California, United States of America; 5 Institute of Neuroscience, Chinese Academy of Sciences, Shanghai, China; K.U.Leuven, Belgium

## Abstract

As in many species, gustatory pheromones regulate the mating behavior of *Drosophila*. Recently, several *ppk* genes, encoding ion channel subunits of the DEG/ENaC family, have been implicated in this process, leading to the identification of gustatory neurons that detect specific pheromones. In a subset of taste hairs on the legs of *Drosophila*, there are two *ppk23*-expressing, pheromone-sensing neurons with complementary response profiles; one neuron detects female pheromones that stimulate male courtship, the other detects male pheromones that inhibit male-male courtship. In contrast to *ppk23*, *ppk25*, is only expressed in a single gustatory neuron per taste hair, and males with impaired *ppk25* function court females at reduced rates but do not display abnormal courtship of other males. These findings raised the possibility that *ppk25* expression defines a subset of pheromone-sensing neurons. Here we show that *ppk25* is expressed and functions in neurons that detect female-specific pheromones and mediates their stimulatory effect on male courtship. Furthermore, the role of *ppk25* and *ppk25*-expressing neurons is not restricted to responses to female-specific pheromones. *ppk25* is also required in the same subset of neurons for stimulation of male courtship by young males, males of the *Tai2* strain, and by synthetic 7-pentacosene (7-P), a hydrocarbon normally found at low levels in both males and females. Finally, we unexpectedly find that, in females, *ppk25* and *ppk25*-expressing cells regulate receptivity to mating. In the absence of the third antennal segment, which has both olfactory and auditory functions, mutations in *ppk25* or silencing of *ppk25*-expressing neurons block female receptivity to males. Together these results indicate that *ppk25* identifies a functionally specialized subset of pheromone-sensing neurons. While *ppk25* neurons are required for the responses to multiple pheromones, in both males and females these neurons are specifically involved in stimulating courtship and mating.

## Author Summary

*Drosophila* mating behaviors serve as an attractive model to understand how external sensory cues are detected and used to generate appropriate behavioral responses. Pheromones present on the cuticle of *Drosophila* have important roles in stimulating male courtship toward females and inhibiting male courtship directed at other males. Recently, stimulatory pheromones emitted by females and inhibitory pheromones emitted by males have been shown to stimulate distinct subsets of gustatory neurons on the legs. We have previously shown that a DEG/ENaC ion channel subunit, *ppk25*, is involved in male courtship toward females but not in inhibition of male-male courtship. Here we show that *ppk25* is specifically expressed and functions in a subset of gustatory neurons that mediate physiological and behavioral responses to female-specific stimulatory pheromones. Furthermore, *ppk25* is also required for the function of those neurons to activate male courtship in response to other pheromones that are not female-specific. In addition to their roles in males, we find that *ppk25*, and the related DEG/ENaC subunits *ppk23* and *ppk29*, also stimulate female mating behavior. In conclusion, these results show that, in both sexes, *ppk25* functions in a group of neurons with a specialized role in stimulating mating behaviors.

## Introduction

Ever since the identification of the first pheromone, *Bombykol*, as the sexual attractant of the silkmoth more than fifty years ago [1], the mechanisms underlying the detection of pheromones and their regulation of animal behavior have been an important area of inquiry. The expanding understanding of the molecular basis of pheromone detection has been aided by studies in *Drosophila melanogaster*, a species in which males perform a series of highly stereotyped behaviors toward females eventually culminating in mating [2], [3]. A number of pheromones that modulate male courtship have been identified [4], [5]. 7,11-heptacosadiene (7,11- HD) and 7,11-nonacosadiene (7,11-ND) are the major excitatory compounds selectively produced by mature females, while 7-Tricosene (7-T) and the volatile cis-Vaccenyl acetate (cVA) are produced by mature males and inhibit male-male courtship. While olfaction is involved in the inhibition of courtship by cVA (reviewed in [6]) and stimulation of courtship by unknown fly odors [7], as well as by food odors [8], most known *Drosophila* pheromones are low volatility cuticular hydrocarbons believed to be detected by direct contact with gustatory organs [9].

Recently, several laboratories have independently reported that three members of the DEG/ENaC family of ion channel subunits, *ppk25*, *ppk23* and *ppk29*, are required for the gustatory detection of pheromones that modulate male courtship behavior [10]–[14]. *ppk23* expression marks a subset of gustatory neurons, two per chemosensory hair, both of which also express *fruitless* (*fru*), a key transcription factor that regulates sexually dimorphic development of neurons involved in sex-specific behaviors (reviewed in [15]). Importantly, *ppk23*-expressing cells respond to pheromones [13], [14], and the two cells present in a single chemosensory hair detect distinct compounds [13]. F cells (female-sensing) respond to female stimulatory pheromones, while M cells (male-sensing) respond to inhibitory male pheromones. *ppk25* is also required for courtship behavior, but unlike *ppk23*, *ppk25* is only expressed in one *fru*-positive gustatory neuron per chemosensory bristle. Furthermore, while loss of *ppk23* function decreases courtship of females and also increases courtship directed at other males [13], [14], mutations in *ppk25* decrease male courtship of females, but do not increase courtship of other males [10], [16]. Together, these results raised the possibility that *ppk25* neurons represent a functionally specialized subset of pheromone-sensing neurons [17].

Here, we show that *ppk25* specifically marks the F cell subset of *ppk23*-expressing cells and is required for their response to stimulatory female-specific pheromones. In contrast, *ppk23*-expressing M cells do not express *ppk25* or require *ppk25* function to detect inhibitory male pheromones. Furthermore, we show that the function of *ppk25* is not restricted to gustatory detection of female-specific pheromones. *ppk25* and *ppk25*-expressing neurons are also required for stimulation of courtship by pheromones present on immature males [18], and by pheromones present on males of the Tai2 strain [19]. Finally, we show that in addition to regulating male courtship behavior, *ppk25* and *ppk25*-expressing gustatory neurons also regulate female mating behavior, suggesting their involvement in detection of male pheromones that stimulate female receptivity.

## Results

### Sensory neurons expressing *ppk25* detect female pheromones that stimulate male courtship

**Figure 1:**
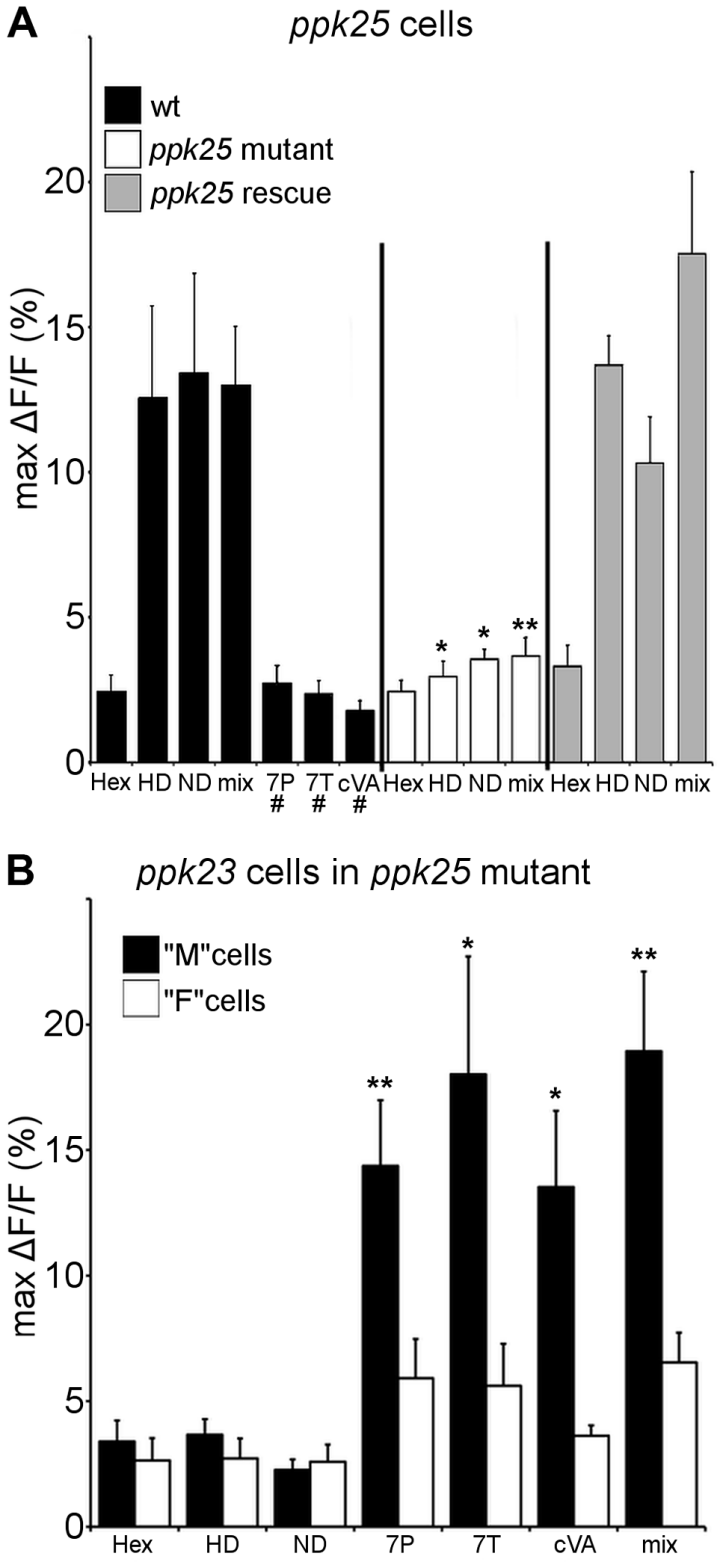
Calcium imaging reveals that *ppk25* cells respond specifically to female pheromones. A. Solutions (100 ng/µl in 10%hexane, 90% water) of 7,11-HD (HD), 7,11-ND (ND), 7T, cVA, a mixture of all pheromones (mix) or 10% hexane, 90% water solution alone (hex) were applied to single leg bristles of *ppk25-Gal4*, *20×UAS-GCaMP3* flies. “*wt*” flies contained one copy of the normal *ppk25* gene. *ppk25 null* mutants were heterozygous for two different deletions of the *ppk25* locus [16], and “*ppk25* rescue” flies are *ppk25* mutants carrying *UAS-ppk25* and *ppk25-Gal4* transgenes to target *ppk25* expression to *ppk25* cells [10]. “#” denotes pheromones that did not elicit responses significantly higher than hexane alone in “*wt*” flies and were not tested further. n = 7–10; Mean ± SEM; ttest to *wt*, *p<0.05, **p<0.01. B. The same pheromone solutions as in A were applied to single leg bristles of *ppk25* mutants carrying the *ppk23-Gal4* and *20×UAS-GCaMP3* transgenes. As previously observed in flies with normal *ppk25* genes [13], one population of *ppk23* cells, the M cells, respond specifically to male pheromones. In contrast to *wt* males however, in *ppk25 null* mutants the second population of *ppk23* cells, corresponding to F cells does not respond to any pheromone. n = 8; Mean ± SEM; ttest to *wt*,*p<0.05,**p<0.01.

To evaluate the ligand specificity of *ppk25* cells, expression of the genetically-encoded calcium indicator, G-CaMP3 [20], was targeted using the *ppk25-Gal4* driver [10]. Single bristles on the front legs of both males and females were stimulated with two female pheromones that had been previously shown to stimulate the F (female-sensing) subset of *ppk23*-expressing cells (7,11-HD and 7,11-ND), and three compounds produced by males that stimulate M (male-sensing) cells (7-tricosene (7T), 7-Pentacosene (7P) and cVA) [13]. As shown previously for F cells [13], *ppk25*-expressing cells in both males and females showed robust calcium responses to the female pheromones, 7,11-HD and 7,11-ND, but not to the male compounds, 7T, 7P or cVA (Fig. 1A). Importantly, this response requires *ppk25*, as *ppk25* null mutants no longer responded to the female cues and targeted expression of *ppk25* in mutants rescued this defect (Fig. 1A). To confirm that *ppk25* is required in cells that detect female pheromones but not in those that detect male pheromones, *ppk23-Gal4* was used to drive expression of G-CaMP3 in all pheromone-sensing cells in a *ppk25* null mutant background. As described previously for flies with normal *ppk25* [13], *ppk23-Gal4* labeled two cells under each bristle in *ppk25* mutants. However, while one of these cells responded specifically to male compounds as previously described for M cells [13], the second cell, did not respond to female compounds as expected of F cells (Fig. 1B). Thus, *ppk25* is essential for the recognition of courtship-stimulating pheromones produced by females but not of courtship-inhibiting pheromones produced by males.

**Figure 2:**
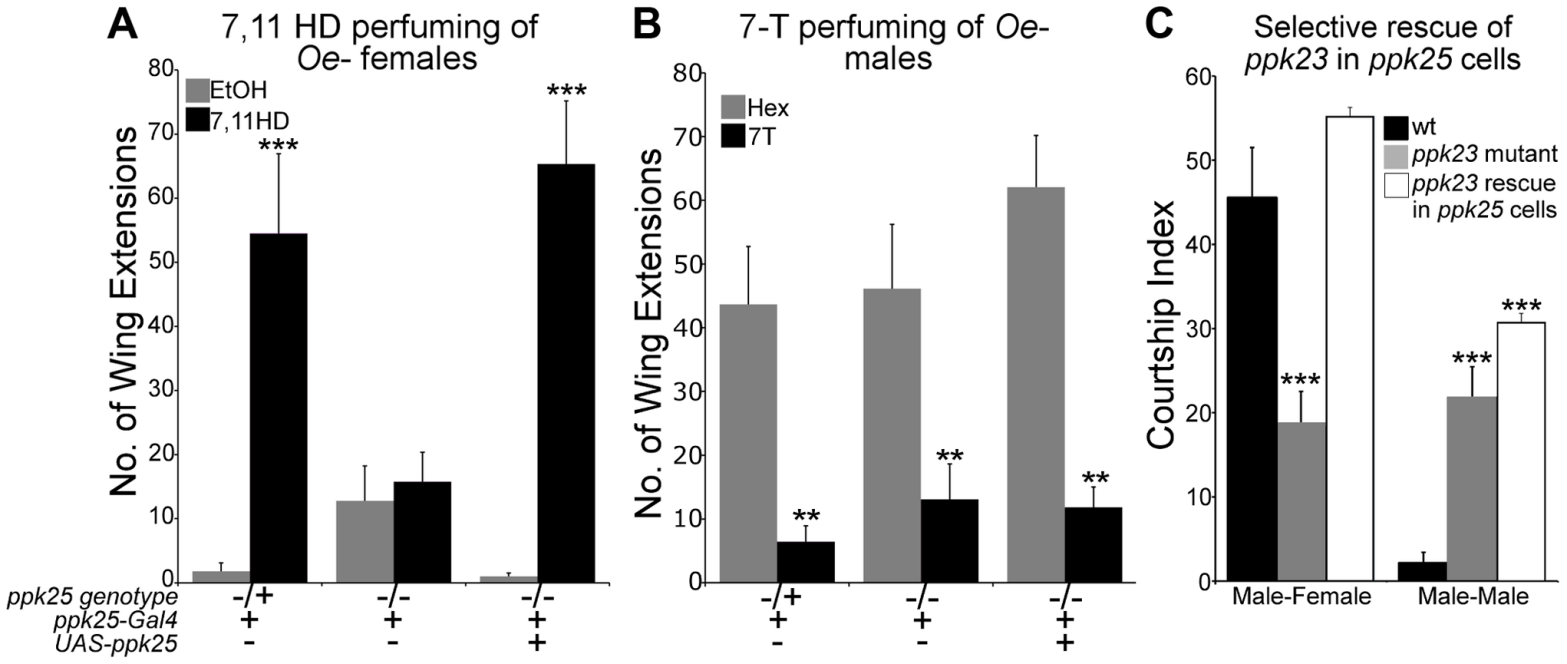
*ppk25* is required for courtship stimulation by 7,11HD but not for inhibition of courtship by 7-T. A. The number of wing extensions performed by males with normal or mutant copies of *ppk25*, or by *ppk25* mutant males in which *ppk25* function is restored specifically in *ppk25* cells, was measured in the presence of oe- females painted either with solvent alone or with a single female pheromone, 7,11HD. oe- females were pierced through the head with forceps, contributing to the low courtship background. These and all following assays involving perfumed oe- targets were conducted under normal laboratory lights [13]. n = 19–24; Mean ± SEM; ***p<0.001 (perfumed relative to solvent control). Error bars indicate the SEM for number of wing extensions and statistical significance was determined using the Kruskal-Wallis test followed by Dunn's post hoc test. B. The number of wing extensions displayed by males with normal or mutant *ppk25* was measured in the presence of oe- males painted either with solvent alone or with a single male pheromone, 7T. In this experiment, males were raised in groups as this resulted in higher baseline courtship toward oe- targets, thereby allowing inhibition to be measured. n = 24–28; Mean ± SEM; **p<0.01; (Kruskal-Wallis test followed by Dunn's post hoc test to solvent control). C. The Courtship Index (CI, percentage of a ten minute observation time during which the male is courting [16]) was measured for control *w1118* males, males mutant for *ppk23*, and *ppk23* mutant males in which expression of *ppk23* is targeted to F cells using the *ppk25-Gal4* driver. Male-female courtship was measured in the presence of decapitated w1118 females to reduce behavioral feedback. Male-male courtship was performed with intact male targets in the light since *ppk23* mutants display robust male-male courtship under these conditions [13], [14]. n = 30–40; Mean ±SEM; ***p<0.001 to *wt*. Error bars indicate the SEM for CI and statistical significance was determined using the Kruskal-Wallis test followed by Dunn's post hoc test.

To test whether *ppk25* is essential for behavioral responses to individual pheromones, control and *ppk25* mutant males were paired with oenocyte-lacking (oe-) flies painted with single cuticular hydrocarbons in courtship assays. In oe- flies, the pheromone-producing cells called oenocytes have been genetically ablated, such that only residual hydrocarbons are present on their cuticle. oe- individuals therefore serve as pheromone-blank flies to which a single synthetic pheromone can be added to test its effect on male courtship [21]. For better comparison with previous studies on the effects of *ppk23* mutations [13], the number of wing extensions in a 20-minute observation period was used as a measure of male courtship. As observed previously [13], painting oe- females with 7,11 HD, the excitatory female-specific pheromone, increased the levels of courtship displayed by control males. In contrast, *ppk25* mutant males were not affected by the presence of 7,11 HD, and this stimulatory effect was restored by targeted expression of *ppk25* (Fig. 2A). In contrast to the inability of *ppk25* mutants to detect stimulatory female pheromones, painting oe- males with 7-T inhibited wing extensions of *ppk25* mutant and normal males to similar extents (Fig. 2B). Thus, *ppk25* mutant males respond to male cuticular hydrocarbons but behave as though they are selectively blind to female cuticular hydrocarbons. These data demonstrate that *ppk25* is specifically required for the stimulatory effect of 7,11 HD, the major female taste pheromone, but is not required for detection of the major male inhibitory taste pheromone, 7T.

In addition to decreasing male courtship toward females, loss of *ppk23* also increases male courtship toward other males and both phenotypes are efficiently rescued by expressing *ppk23* in *ppk23-Gal4* cells [13], [14]. To test whether the *ppk25*-positive cells are involved in both courtship defects of *ppk23* mutants, we rescued *ppk23* function exclusively in *ppk25-Gal4* cells in a *ppk23* mutant background. Male flies expressing functional *ppk23* only in *ppk25* cells showed normal courtship toward females but, like *ppk23* mutant males, displayed abnormally high levels of male-male courtship as measured using the courtship index (CI, the percentage of a ten minute observation time during which the male is courting [16]) (Fig. 2C). These experiments show that *ppk25* cells are required for normal recognition of female-specific pheromones and for their stimulatory effects on male courtship whereas the *ppk23*-positive, *ppk25*-negative cells are required for recognition of inhibitory male pheromones.

### *ppk25* is required for detection of courtship-stimulating pheromones present in young males and *Tai2* males

**Figure 3:**
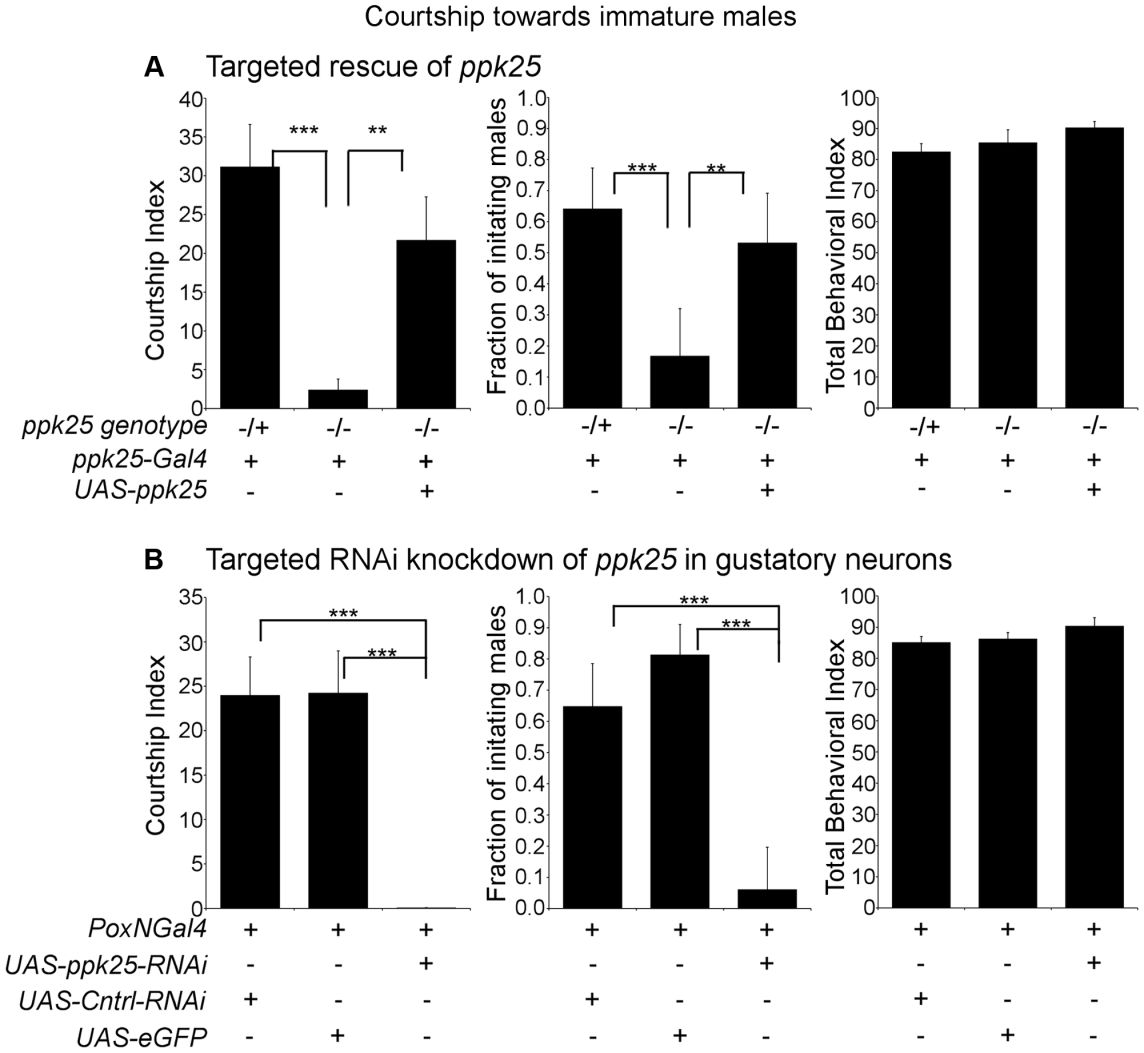
Male courtship of immature males requires *ppk25* function. A. *ppk25* expression in gustatory neurons defined by *ppk25-Gal4* is required for male courtship of immature males. CI, fraction of males initiating courtship toward decapitated immature males and Total Behavioral Index (TBI) were calculated for *ppk25* mutant males, control males with one *wt* copy of *ppk25* and for *ppk25 null* mutant males where *ppk25* expression has been restored in cells expressing *ppk25-Gal4*. N = 32–39; Mean ± SEM; ***p<0.001; **p<0.01 (Error bars indicate the SEM for the fraction of males initiating courtship and statistical significance was determined by Fisher's exact test. Error bars and statistical significance for TBI was determined as described previously for CI). Newly eclosed Canton-S males with light body pigmentation and unfurled wings were used as immature male targets. B. Targeted RNAi knockdown of *ppk25* in gustatory neurons with *Poxn-Gal4* reduces courtship toward *Tai2* males. CI, fraction of males initiating courtship toward decapitated *Tai2* males and TBI were calculated for males expressing *ppk25* RNAi in gustatory neurons. Control males expressed eGFP or a control RNAi targeting *CG13895*, a gene with no known involvement in mating behavior [58] in all gustatory neuron. N = 32–34; Mean ± SEM; ***p<0.001.

Given the requirement of *ppk25* for detection of female stimulatory pheromones but not male inhibitory pheromones, we investigated whether *ppk25* might also be required for detection of stimulatory pheromones that are not specific to the female. Immature *Drosophila* of either sex, while lacking adult female-specific pheromones, efficiently elicit courtship from adult males, most likely as a result of the unusual long-chain hydrocarbons present on their cuticle [18]. We therefore tested whether *ppk25* is needed for male courtship of immature males (Fig. 3). As expected, males with one functional copy of *ppk25* showed strong courtship toward young males. In contrast, *ppk25* homozygous mutant males performed very little courtship as measured by either CI or fraction of males initiating courtship, but courtship was restored in *ppk25* mutants when *ppk25* expression was driven with the *ppk25-Gal4* driver (Fig. 3A). As shown previously [10], mutations in *ppk25* do not cause a generalized defect in behavior since the Total Behavior Index (TBI - the fraction of the observation time the male spends performing any observable behavior: courtship, walking or preening) was not affected in *ppk25* mutants (Fig. 3A).

To further test the role of *ppk25* in courtship of immature males and to identify the type of cell involved, we knocked-down *ppk25* mRNA using targeted expression of a *UAS-ppk25-RNAi* transgene [10] with *Poxn-Gal4*, a driver expressed specifically and at high levels in all gustatory neurons [22]. In control males, *Poxn-Gal4* drove expression of either GFP, or of an RNAi that targets an unrelated gene (*CG13895*). Compared to control males, males with gustatory neuron-specific knockdown of *ppk25* courted young males at significantly reduced levels, as reflected by a reduction in both CI and fraction of initiating males, while the TBI remained unchanged (Fig. 3B). Together, these results indicate that *ppk25* function in gustatory neurons marked by expression of *ppk25-Gal4*, is required for activation of courtship not only by female-specific pheromones [10], but also by pheromones present on immature males.

**Figure 4:**
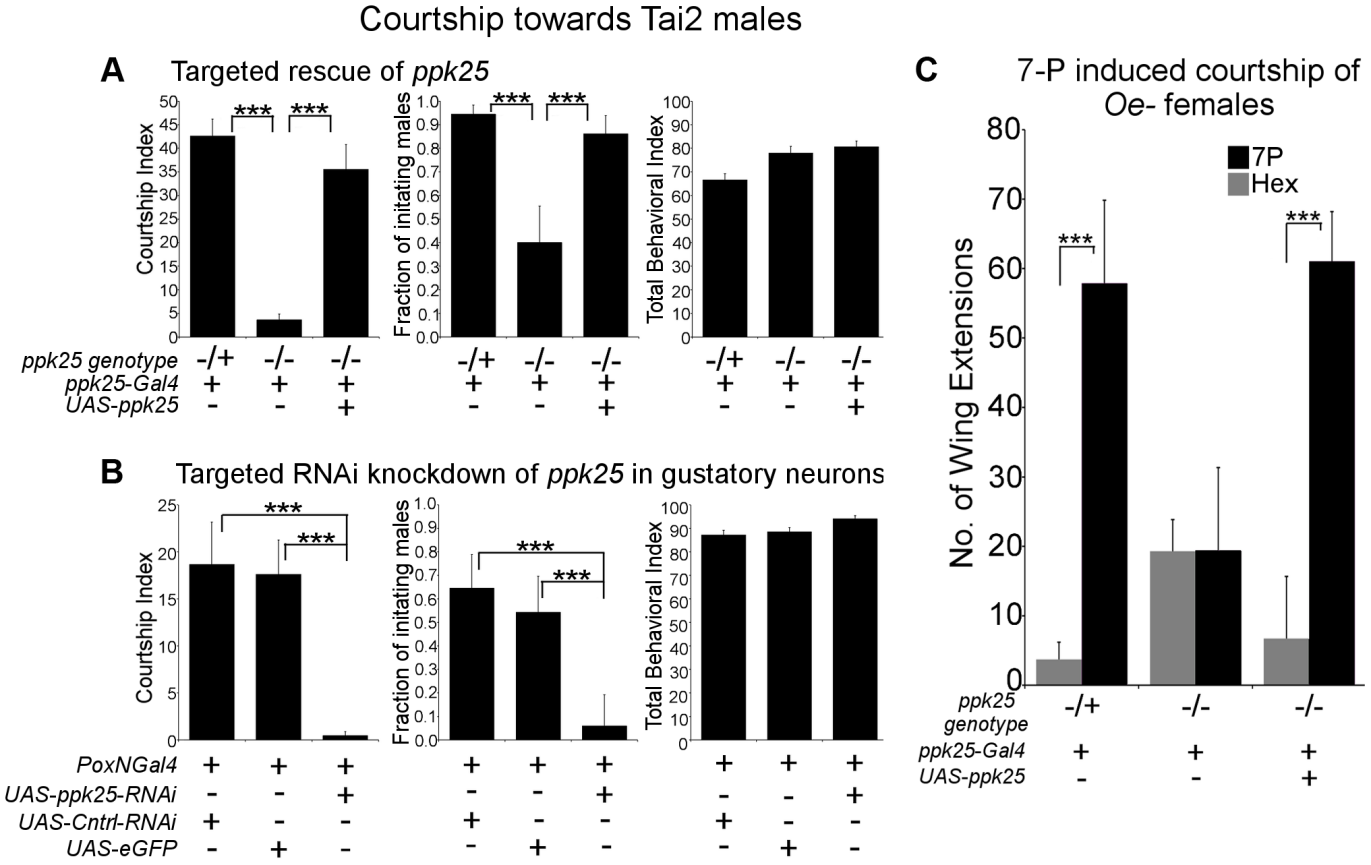
*ppk25* function in gustatory neurons is required for courtship stimulation by *Tai2* males or 7-pentacosene. A. *ppk25* function in cells defined by *ppk25-Gal4* expression is required for male courtship directed at *Tai2* males. CI, fraction of males initiating courtship toward decapitated *Tai2* males and TBI were calculated for *ppk25 null* mutant males, control males with one wild-type copy of *ppk25*, and *ppk25* mutant males where *ppk25* expression had been restored in *ppk25* cells. N = 36–40; Mean ± SEM; ***p<0.001. B. Targeted RNAi knockdown of *ppk25* in all gustatory neurons reduces courtship toward *Tai2* males. CI, fraction of males initiating courtship toward decapitated *Tai2* males and TBI were calculated for males expressing *ppk25* RNAi in gustatory neurons using *Poxn-Gal4*. Control males expressed eGFP or a control RNAi targeting *CG13895* under control of *Poxn-Gal4*. Error bars are SEM; N = 31–34; ***p<0.001. C. The number of wing extensions performed by males with normal or mutant copies of *ppk25*, or by *ppk25* mutant males in which *ppk25* function is restored in *ppk25* cells, was measured in the presence of oe- females painted either with solvent alone or with 7-Pentacosene. oe- females were pierced through the head with forceps, contributing to the low courtship background and assays were conducted in the light. n = 20–24; Mean ± SEM; ***p<0.001.

In addition to females and immature males, males of the naturally occurring Tai2 strain of *Drosophila melanogaster* also stimulate male courtship, likely as a result of their unusual pheromone profile [19], [23]. As expected, control males displayed high levels of male-male courtship when paired with Tai2 males (Fig. 4A). In contrast, courtship of Tai2 males was largely eliminated in *ppk25* mutant males and rescued by targeted expression of *ppk25* using *ppk25-Gal4* (Fig. 4A). Finally, knockdown of *ppk25* in all gustatory neurons using *Poxn-Gal4*-driven RNAi results in a similarly severe decrease in male courtship of Tai2 males (Fig. 4B). Together, these results show that courtship stimulation by Tai2 males requires *ppk25* in the subset of gustatory neurons defined by expression of *ppk25-Gal4*.

One of the significant differences in the pheromone profile of Tai2 males is the elevated level of 7-Pentacosene (7P) [19], [23], which can stimulate male courtship behavior [24] and may therefore underlie courtship stimulation by Tai2 males. We therefore tested whether *ppk25* is required for the stimulation of courtship by 7-P. 7-P perfuming of oe- females resulted in a significant increase in the wing extensions for control males, but not for males lacking functional *ppk25*, and the stimulatory effect of 7-P was restored in *ppk25* mutants by targeted expression of *ppk25* with *ppk25-Gal4* (Fig. 4C). These results show that, as is the case for 7,11-HD (Fig. 2), the stimulatory effect of 7-P on male courtship requires *ppk25* function in *ppk25-Gal4* cells.

Thus, in addition to functioning in the detection of female-specific pheromones that stimulate male courtship, *ppk25* and *ppk25*-expressing neurons are important for detecting at least two other types of excitatory pheromonal cues that promote courtship: pheromones emitted by immature Drosophila, and 7-P, likely accounting for its requirement in the courtship of Tai2 males. In contrast, and unlike *ppk23, ppk25* is not required for detection of the major male inhibitory pheromone, 7T, indicating that *ppk25*-expressing neurons represent a subset of pheromone-sensing neurons specialized in detecting pheromones that stimulate male courtship behavior.

### *ppk25* is involved in female receptivity

**Figure 5:**
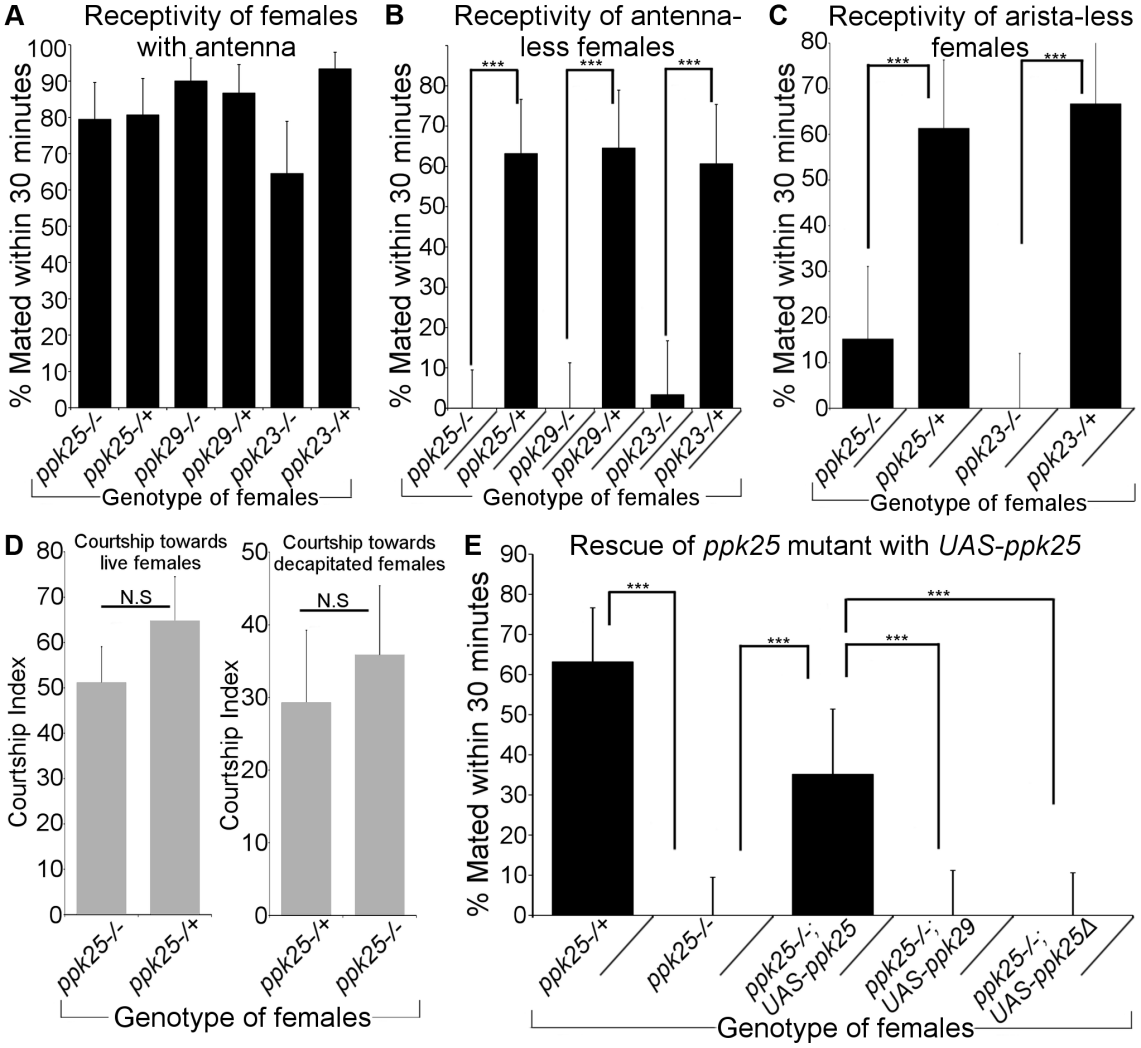
Antenna-less females mutant for *ppk25, ppk23 or ppk29* are unreceptive to males. A. The percentage of females that mated within 30(“Receptivity”) was calculated for females with homozygous null mutations in *ppk25*, *ppk23* or *ppk29* [11], [13], [16], and for control females with one normal copy of *ppk25*, *ppk23* and *ppk29*. N = 30–34. For receptivity in Fig. 5 and 6, error bars indicate the SEM. B. Antenna-less females mutant for *ppk25*, *ppk23 or ppk29* show reduced receptivity to males. Receptivity was measured for mutants and control females as in A but after removal of antennae. N = 30–38; ***p<0.001. (Fisher's exact test). C. Arista-less females mutant for *ppk23 or ppk25* show reduced receptivity to males. Receptivity was measured for mutants and control females as in A but after removal of arista. N = 21–32; ***p<0.001. (Fisher's exact test). D. *wt* males find *ppk25* mutant and control females equally attractive. CI measured for Canton-S males toward females that were either alive (left) or decapitated (right). CI of males toward live females was quantified by measuring male courtship behavior during the first 10 minutes of the receptivity assay shown in Fig. 5B. Females were either homozygous null mutants for *ppk25* or contained one normal copy of *ppk25*. N = 20. E. Introduction of a *ppk25* transgene increases receptivity of *ppk25* mutants but not of *ppk29* mutants. Receptivity was measured for antenna-less females carrying an *UAS-ppk25* transgene in either a *ppk25* mutant or *ppk29* mutant background and compared to *ppk25* mutant and controls shown earlier (panel A). N = 30–38; ***p<0.001 (Fisher's exact test).

Since *ppk25* is required for the detection of a variety of stimulatory pheromones in males, we considered the possibility that it may also be involved in regulating female behavior. Indeed, expression of reporters under the control of *ppk25-Gal4* [10], as well as *ppk23-Gal4* and *ppk29-Gal4* [11]–[14] is seen in chemosensory neurons of females. Furthermore, chemical senses, in addition to vision and hearing, likely control female receptivity to mating [25]–[27]. To test the role of DEG/ENaC channels in regulating female receptivity, we paired wild-type (Canton-S) males with control females or females that were homozygous mutant for *ppk23*, *ppk25* or *ppk29*, and used the fraction of females that mated within thirty minutes as a measure of female receptivity [28]. Females with homozygous mutations in *ppk25*, *ppk23* or *ppk29* displayed showed similar levels of receptivity as control females (Fig. 5A). However, we considered the possibility that the apparent lack of requirement for *ppk25*, *ppk23* and *ppk29* in female receptivity observed under our experimental conditions could result from the redundant action of multiple sensory cues. Indeed, olfactory and/or acoustic signals detected by the antennae play a crucial role in controlling female receptivity [25], [26], [29], [30]. We therefore tested the effect of *ppk* mutations on the receptivity of females whose antennae had been inactivated by surgical removal of the third antennal segment. Even after antennal inactivation, more than 60% of control females mated within 30 minutes. In sharp contrast, females that were homozygous mutant for *ppk25*, *ppk29* or *ppk23* displayed little, if any, receptivity (Fig. 5B). In order to further explore the redundant antennal cues driving receptivity, we looked at the effect of *ppk25* and *ppk23* mutations on the receptivity of females that were only lacking the aristae rather than the whole antennae, thereby impairing auditory function but leaving olfactory function intact [31]. Here again, we find that while the receptivity of control females remains high after removal of the arista, mutations in *ppk23* and *ppk25* significantly decrease receptivity in aristaless females (Fig. 5C). These data suggest that acoustic stimuli are sufficient to promote high levels of female receptivity in the absence of functional *ppk25* or *ppk23*. The effect of *ppk25* inactivation on mating does not result from a reduced sexual attractiveness of the mutant females, as levels of male courtship during these female receptivity assays are similar in the presence of control and *ppk25* mutant females (Fig. 5D, left panel). To further rule out any possible effect of the female genotype on male behavior, we conducted male courtship assays using decapitated *ppk25* mutant females and controls, thereby reducing behavioral feedback from females. In these assays, males courted both sets of females with equal intensity (Fig. 5D, right panel).

Surprisingly, given that the *UAS-ppk25* transgene by itself does not rescue the courtship defect of *ppk25* mutant males [10], we find that the presence of *UAS-ppk25* significantly increases the receptivity of *ppk25* mutant females (Fig. 5E). This observation suggests that leaky expression from *UAS-ppk25*, as seen with other *UAS* transgenes [11], [32], [33], is sufficient to partially rescue *ppk25* function in females, further supporting the role of *ppk25* in female receptivity. Indeed, a similar transgene with a truncated version of *ppk25*, *UAS-ppk25Δ* [10], or a *UAS-ppk29* rescue transgene [11] do not increase the receptivity of *ppk25* mutant females (Fig. 5E), and the *UAS-ppk25* transgene does not increase the receptivity of *ppk29* mutant females (data not shown). Taken together, these results suggest that all three DEG/ENaC genes play critical roles in female receptivity that can be obscured by the presence of redundant sensory inputs from the antennae.

**Figure 6:**
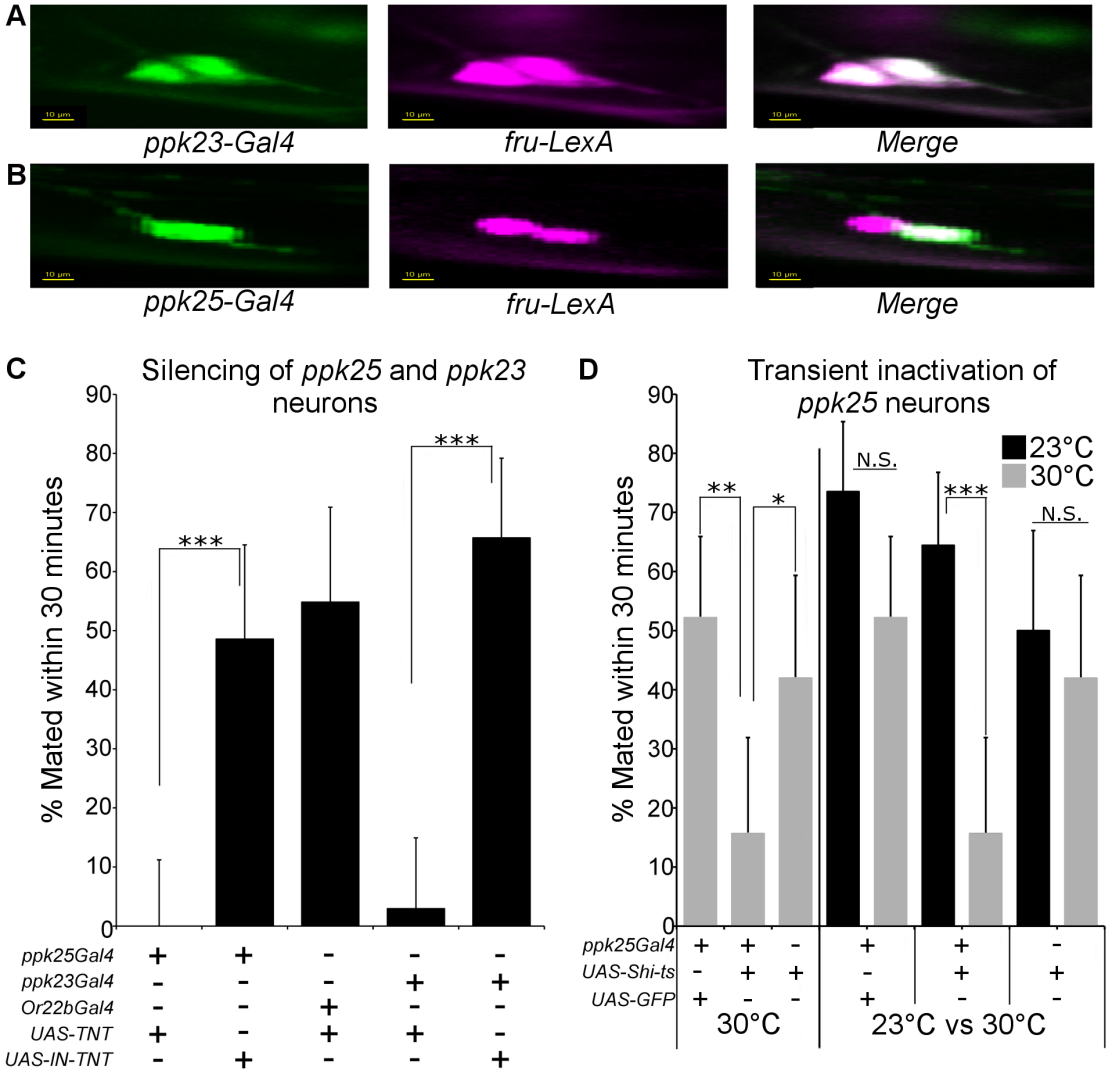
Female *ppk25-Gal4* neurons represent a subset of *ppk23-Gal4* neurons and are involved in female receptivity. A. Segment of female front legs showing expression of *ppk23-Gal4* (green) in each of the two *fru*-positive cells (*fru-LexA*, magenta) under each chemosensory bristle. Scale 10 µ*m*. B. Segment of female front legs showing expression of *ppk25-Gal4* (green) in only one of the two *fru*-positive cells (*fru-LexA*, magenta) present under each chemosensory bristle. Scale 10 µ*m*. C. Silencing of *ppk25-Gal4* or *ppk23-Gal4* neurons reduces female receptivity. Receptivity was calculated for females expressing active (*UAS-TNT*) or inactive (*UAS-IN-TNT*) forms of TNT under control of either *ppk23-Gal4* or *ppk25-Gal4*. Additional control females contained *UAS-TNT* driven by *Or22b-Gal4*, which is expressed in a neuronal subset not associated with mating behaviors [36]. N = 30–35; ***p<0.001 (Fisher's exact test). D. Transient inactivation of *ppk25-Gal4* neurons inhibits female receptivity. Females expressing the temperature-sensitive dominant *Dynamin* allele *shi^ts^* [37] under control of *ppk25-Gal4* were incubated at either permissive (23°C) or non-permissive (30°C) temperature for 20 minutes prior to, and during the test. Receptivity for females expressing GFP in *ppk25-Gal4* neurons or females with *UAS-Shi^(ts)^* alone was measured under identical conditions. N = 30–45; ***p<0.001;**p<0.01;*p<0.05 (Fisher's exact test).

Since in males, *ppk25* and *ppk23* function within defined subsets of gustatory neurons [10]–[14], we sought to clarify the relationships between the expression of these *ppks* in female gustatory neurons, and to test their function in regulating female receptivity. Expression of *ppk25* and *ppk23* in females was visualized by driving expression of GFP using *ppk25-Gal4* and *ppk23-Gal4*. Consistent with the sexual dimorphism in the total number of taste hairs on the front legs [34] as well as in the number of *ppk23*-expressing taste hairs [12], [13], there are fewer *ppk25*-expressing hairs on the front legs of females compared to males (data not shown). As seen in males [12]–[14], *ppk23*-positive hairs have two *fru*-positive chemosensory neurons labeled by *fru*-*LexA*-driven expression of Red Fluorescent Protein (RFP), both of which also express GFP under the control of *ppk23-Gal4* (Fig. 6A). In contrast, *ppk25-Gal4*-driven expression of GFP co-localizes with only one of the two *fru*-positive cells within any particular taste hair (Fig. 6B). Therefore, as in males, *ppk23-Gal4* is expressed in each of the two *fru*-positive taste neurons present in a subset of chemosensory bristles, one of which also expresses *ppk25-Gal4*.

To test the function of neurons expressing *ppk25* or *ppk23*, we used the same drivers to target expression of tetanus toxin (TNT), a synaptic transmission blocker [35]. For both *ppk25-Gal4* and *ppk23-Gal4*, targeted expression of TNT, but not of an inactive variant (IN-TNT), resulted in a severe loss of receptivity. (Fig. 6C). In contrast, expression of TNT within *Or22b*-expressing olfactory neurons that have no known role in taste or pheromone reception [36], had no effect. To discriminate between developmental and acute requirements of neuronal function, we expressed the temperature-sensitive version of *Drosophila* dynamin, *shibire* (*Shi^ts^*) [37] in *ppk25-Gal4* neurons and shifted females to the restrictive temperature of 30°C a few minutes before and during the receptivity assay. When assayed at 30°C, females expressing *Shi^ts^* in *ppk25*-*Gal4* neurons were significantly less receptive than females expressing GFP in the same neurons or females carrying *UAS-Shi^ts^* but lacking the *Gal4* driver (Fig. 6D). Furthermore, *ppk25-Gal4>UAS-Shi^ts^* females placed at the permissive temperature of 23°C had significantly higher receptivity compared to genetically identical females maintained at the non-permissive temperature (Fig. 6D). Together, these data suggest that the cells identified by *ppk25*- and *ppk23-Gal4* expression are directly involved in female receptivity to mating, most likely in response to male pheromones.

## Discussion

### Expression and function of *ppk25* define a subset of gustatory neurons that stimulate male courtship in response to a variety of pheromones

GCaMP-based calcium imaging of *ppk23*-expressing, pheromone-sensing gustatory neurons identified two types of neurons with different response specificities: F cells respond to female-specific pheromones that stimulate male courtship, while M cells respond to pheromones enriched in males that inhibit male courtship [13]. Here, we present several lines of evidence indicating that *ppk25* expression specifically labels F cells. First, GCaMP imaging shows that *ppk25*-expressing cells respond to female-specific stimulatory pheromones but not to male inhibitory pheromones. Second, in *ppk25* mutants, F cells lose their response to pheromones while the response of M cells is unchanged. Finally, behavioral responses to 7,11HD, a female-specific pheromone detected by F cells, requires *ppk25* function in *ppk25-Gal4* neurons, while behavioral response to 7T, a male inhibitory pheromone detected by M cells, does not. These findings are also consistent with previous results showing that *ppk25* is required for stimulation of male courtship by females but not for inhibition of male-male courtship [10]. Together, these observations identify a functionally specialized group of gustatory neurons defined by *ppk25* function and expression that detects female-specific pheromones and mediates their stimulatory effect on male courtship. Furthermore, mutations in *ppk23* or silencing of *ppk23* neurons result in loss of both the physiological and behavioral effects of male inhibitory pheromones [13], [14], while mutations in *ppk25* or silencing of *ppk25* neurons do not ([10] and this report). Consistent with those observations, we find that *ppk23* mutant males in which *ppk23* function is rescued specifically in *ppk25* neurons have normal responses to females but still display increased courtship of other males. Together, these data suggest that pheromone-sensing neurons fall into two functionally complementary types. F cells are defined by expression of *ppk25*, respond to female-specific pheromones, and mediate the stimulatory effects of those pheromones on courtship. In contrast, M cells express *ppk23* but not *ppk25*, respond to male pheromones, and mediate the inhibitory effects of those pheromones on courtship.

In addition to female-specific pheromones, pheromones found on immature *Drosophila* [18] as well as 7-Pentacosene [24] can activate male courtship. Both targeted rescue and knockdown experiments demonstrate that the activation of courtship by these pheromones or pheromone blends requires functional *ppk25* within the subset of gustatory neurons that also mediate responses to adult female pheromones ([10] and this report). The requirement of *ppk25* cells for courtship activation by 7P was surprising given that, in both males or females, while 7P elicits a response from cells that respond to male inhibitory pheromones ([13] and this report), *ppk25* cells display no detectable response to this hydrocarbon (Fig. 1). Compared to their responses to female-specific pheromones, *ppk25* cells may respond to 7P with either lower intensity or slower kinetics, preventing detection in our GCaMP assay. Indeed, the physiological responses of olfactory neurons to their cognate olfactory neurons vary in both magnitude and kinetics, neither of which correlates with the strength of the behavioral response [38]. Furthermore, the volatile pheromone cVA was originally shown to strongly activate *Or67d* neurons [39] but an improved odor testing paradigm revealed that cVA also activates *Or65a* neurons weakly [40] and subsequent work showed that *Or65a* neurons are critical for cVA's chronic effects on *Drosophila* aggression [41]. Finally, while we have tested responses from *ppk25*-expressing cells at different positions on the front legs, we cannot rule out the possibility that a subset of *ppk25* cells, perhaps in a less accessible part of the front leg, or on the second or third pairs of legs, display a detectable GCaMP response to 7P. Alternatively, the lack of GCaMP signal may accurately reflect the fact that *ppk25* neurons do not directly detect 7P. By analogy with the integration of olfactory information resulting from non-synaptic interactions between neighboring olfactory neurons [42], 7-P activation of male courtship may require non-synaptic interactions between *ppk25* cells and the neighboring M cells which detect 7P [13].

Whether it is through the direct detection of pheromones, or in a more indirect regulatory role, *ppk25* neurons are required for the stimulation of courtship by several different pheromones. In contrast, *ppk25* neurons are not required for the responses to at least two different courtship-inhibiting pheromones, suggesting that they are functionally specialized in courtship activation. This specialized function is analogous but opposed to that of the previously described but non-overlapping subsets of neurons on the front legs of males that express *Gr32a* [7], [43], [44] or *Gr66a* [45], [46] and detect pheromones that inhibit male courtship.

### *ppk25* gustatory neurons contribute to redundant sensory inputs that control female receptivity

In addition to their roles in pheromonal control of male courtship, we show here that *ppk25*, *ppk23* and *ppk29* also play critical roles in regulating female mating behavior. During the *Drosophila* courtship ritual, females actively assess the courting male by detecting a variety of cues, including male pheromones and appropriate sensory stimulation of the female is required for mating to occur [47]. Here, we show that *ppk25, ppk23 and ppk29* are all required for antenna-less *Drosophila* females to become receptive to mating. Furthermore, as in males, *ppk23-Gal4* and *ppk25-Gal4* are strongly expressed in gustatory neurons of female legs ([10], [13] and present work). While in addition to its expression in gustatory neurons, *ppk25* is also expressed at lower levels in the olfactory system [10], *ppk23* and *ppk29* are only detectably expressed in gustatory neurons [11]–[14]. Therefore, the dramatic loss of receptivity observed for antenna-less females carrying mutations in any of the three *ppks*, or whose *ppk23-* or *ppk25-*expressing neurons have been silenced suggests that all three *ppks* function in a common subset of pheromone-sensing gustatory neurons that regulate female receptivity to mating. Finally, while the identity of the pheromone(s) involved remains to be determined, the role of *ppk25* in promoting female receptivity is consistent with the involvement of this DEG/ENaC subunit in the responses to multiple pheromones.

These data provide, to the best of our knowledge, the first evidence that female receptivity to mating in *Drosophila* is regulated by gustatory detection of pheromones. Furthermore, gustatory pheromone detection is at least partially redundant with other sensory stimuli, in particular auditory stimuli, as mutations in *ppk* genes only affect the receptivity of females whose aristae have been removed. Similar redundancy exists in sensory stimulation of male courtship behavior in *Drosophila* [9], [16], [44], [48] and, more generally in sensory detection of signals that drive sexual behavior in many other species, with potential evolutionary and ecological advantages [49]. While male pheromones, in particular cVA and 7T, have been shown to regulate female mating receptivity, detection of these two pheromones has been reported to involve olfactory rather than gustatory organs; for cVA through the antennal Or67d olfactory receptor [26] and for 7T through unknown receptors on the antennae [25]. The male pheromone that stimulates female receptivity and is detected by *ppk25* gustatory neurons therefore remains to be identified.

In conclusion, we show that a subset of pheromone-sensing neurons, identified by the expression and function of *ppk25*, have a specialized role in stimulating male courtship and female receptivity in *Drosophila*. The identification and manipulation of these neurons may lead to a better understanding of how gustatory neural circuits drive not only courtship and mating, but also other *Drosophila* behaviors that depend on the detection of conspecific pheromones [50].

Finally, these results are relevant to the consideration of two mutually exclusive models for the molecular role of DEG/ENaC channels in pheromone detection [17]. Channel gating may result from direct interaction with pheromones or pheromones-protein complexes. Alternatively, these channels may have an indirect role, modulating the excitability of specific subsets of pheromone-sensing neurons. The existence of multiple pheromones requiring *ppk25* function is surprising, given the high specificity of known pheromone receptors [51], [52]. However, the three identified *ppk25*-dependent pheromones are unsaturated linear hydrocarbons with chain-lengths varying from C25 to C29 with a double-bond at position C7, while *ppk25* function is not required for 7-T, another hydrocarbon with a double-bond at C7, but with a shorter chain of 23 carbons. The presence of *ppk25* in a heterotrimeric DEG/ENaC could therefore modify channel specificity by requiring a ligand with a longer hydrocarbon chain. However, our results are also compatible with the possibility that *ppk25*, *ppk23* and *ppk29* function less directly by modulating the excitability of pheromone-sensing neurons whose ligand specificity is dictated by as yet unidentified pheromone receptors. The specific function of *ppk25* in pheromone detection should prove invaluable for dissecting the molecular mechanisms underlying the function of *ppk25*, *ppk23* and *ppk29* in pheromone response, with implications for the roles of DEG/ENaCs in a number of other sensory processes [53], [54].

## Materials and Methods

### *Drosophila* stocks

Mutations in *ppk25*, *ppk23* and *ppk29* were described previously [11], [13], [16]. *ppk25* null mutant flies were heterozygous for two different deletions of the *ppk25* gene; an imprecise excision of a *P*-element that removes the regulatory regions and the first half of the *ppk25* gene (Δ*5–22*) and a deletion spanning 20 genes in the *ppk25* regions(Δ*42E*) [16]. *ppk29* mutants were homozygous for a transposable element insertion in exon 5 of the *ppk29* gene [11]. *ppk23* mutants were generated by deletion of an 8.3 kb *ppk23*-containing region through FLP-FRT-mediated recombination of two *piggybac* transposons [13]. Targeted expression of *ppk23* or *ppk25* was achieved using the *ppk25-Gal4*, *UAS-ppk23* and *UAS-ppk25* transgenes [10], [13]. The *UAS-ppk25 RNAi* line was obtained from the Transgenic RNAi Project at Harvard Medical School (stock number JF02434). *Poxn-Gal4*, *fru-LexA*, and *lexAop-FRT-tdTomato::nls; UAS-stinger* lines were gifts from David Mellert and *UAS-Shi^(ts)^* was a gift from Kathy Siwicki. All other lines were obtained from the Bloomington Stock Center at Indiana University.

### G-CaMP imaging

G-CaMP imaging was performed on single leg chemosensory bristles as described previously [13], except for the use of the *20×UAS-GCAMP3* transgene [55]. Briefly, single bristles were stimulated by bringing them in contact with a custom-built glass capillary filled with ∼5 µL of a solution of synthetic pheromones (Cayman Chemical, Ann Harbor, MI). Pheromones were diluted to 100 ng/µl in 10% hexane∶90% water solution. Calcium-induced fluorescent increases of one or two cells under a single bristle were monitored by spinning disk confocal microscopy. Errors bars represent the SEM and t-tests were used for statistical significance.

### Behavioral assays

Flies were raised at 25°C, 50% relative humidity in a 12 h∶12 h light∶dark cycle. For testing male courtship, males were raised individually for 4–8 days post-eclosion unless noted otherwise, while females and males used as courtship targets were raised in groups. In perfuming experiments, synthetic pheromones (Cayman Chemical, Ann Harbor, MI) were applied to oe- males or females lacking cuticular hydrocarbons [21] as described [13]. All assays were conducted under infrared lights and monitored using infrared-sensitive cameras, except perfuming assays with oe- targets and male-male courtship assays, which were performed as previously described [13], [14]. Courtship directed at decapitated females or males with normal cuticular hydrocarbons was measured for 10 minutes and quantitated using the Courtship Index (CI, the fraction of time the male spends performing any courtship behavior ×100 [56]). As a measure of total behavioral activity, we used the Total Behavioral Index (TBI, the fraction of time during which the male courts, walks, or preens ×100 [10]). For better comparison with previous work [13], courtship behavior toward perfumed oe- females and males was measured as the number of times the male extends its wings in a 20-minute observation period. For measures of the number of wing extensions, CI and TBI, error bars indicate the SEM, and the Kruskal-Wallis test followed by Dunn's post hoc test was used to determine statistical significance. For the fraction of males initiating courtship, error bars indicate the SEM, and statistical significance was calculated using Fisher's exact test.

For female receptivity assays, females were aged for 4–8 days before removal of the third antennal segment with fine forceps at least two days before testing. Canton-S males aged 3–6 days were used for all female receptivity assays. Males and females were aspirated into plexiglass chambers, their behavior recorded for 30 minutes in the light and the number of females copulating within 30 minutes was determined. Neuronal inactivation with *Shibire^ts^* was achieved by placing females in a 30° Celsius room for 20 minutes prior to, and during the 30-minute receptivity assay. Error bars for female receptivity indicate the SEM, and statistical significance was calculated using Fisher's exact test. All behaviors were scored blind and analyzed either manually or using the LIFESONG X software (version 0.8) [57].
